# Investigations on Aging Behavior and Mechanism of Polyurea Coating in Marine Atmosphere

**DOI:** 10.3390/ma12213636

**Published:** 2019-11-05

**Authors:** Kaiyuan Che, Ping Lyu, Fei Wan, Mingliang Ma

**Affiliations:** School of Civil Engineering, Qingdao university of technology, Qingdao 266033, China; 18764811850@163.com (K.C.); mamingliang@qut.edu.cn (M.M.)

**Keywords:** polyurea, aging mechanism, morphology, chemical properties, phase separation, hydrogen bond

## Abstract

In this investigation, the aging behaviors of polyurea coating exposed to marine atmosphere for 150 days were studied and the mechanism was analyzed. The influences on surface and mechanical properties, surface morphology, thermal stability behavior, as well as chemical changes evolution of the coating were investigated. By attenuated total reflectance fourier transform infrared spectroscopy (ATR–FTIR) and X-ray photoelectron spectroscopy (XPS), changes in the chemical properties of polyurea coatings before (PCB) and after 150 d (PCA) of aging were analyzed, and emphasis was given to the effect of aging on functional group change, the hydrogen bonding behavior, and phase separated morphology. The results displayed prominent chain scission during aging, such as N–H, C=O, and C–O–C and the hydrogen bonded urea carbonyl content showed a decrease trend. The relative content of soft and hard segments showed a significant change, which increased the degree of phase separation.

## 1. Introduction

Polyurea as a thermoset elastomer has excellent performance for its special phase structure and physical crosslinks, which is formed by reacting a diisocyanate with an amine terminated compound by a step growth polymerization process [1,2,3,4]. Due to its exceptional mechanical and physical properties, chemical and moisture resistance, polyurea has been extensively applied in a wide number of applications. [1,3] For example, polyurea has been found to change the failure and fragmentation behavior upon impact by hypervelocity projectiles and is applied in military fields such as bulletproof vests [5]. In recent years, polyurea has been increasingly used in marine engineering, such as dock steel piles, sea-crossing bridges, and drilling platforms. Polyurea has also been studied as a binder for marine joint structures exposed to impact loads of varying amplitudes and strain rates. Thus, polyurea’s moisture resistance properties and its impact mitigation capacity over a wide range of frequencies make it an ideal candidate for applications that involve exposure to or submergence in sea water [6].

As is known, there are aggressive factors such as ultraviolet radiation, floating and pushing fouling, aggressive chemicals, mechanical stresses, moisture and so on in the ocean atmosphere, which can not only cause failure of coating, but also pose a threat to property and life safety [7,8]. Based on this background, research work on the constitutive properties of polyurea are being given increasingly more interest [9,10,11,12,13]. Youssef [14] investigated the effect of UV radiation on the dynamic mechanical properties of polyurea samples and found that the dynamic creep modulus increased with an increase in the UV exposure duration. Whitten [15] studied the color change from white to deep tan after extended exposure to ultraviolet radiation. Youssef [16] investigated the effect of UV radiation on the ultrasonic properties of polyurea and concluded that the acoustic wave speeds and p-wave attenuation were found to exhibit minimal change, while shear attenuation showed convergence as the temperature and ultraviolet exposure duration increased. Gupta [17] presented a new laser-generated stress-wave-based test method to measure polyurea characteristics under high strain rates and limited strains.

In recent years, many scholars have done a lot of research on the chemical composition of coatings. [18,19,20,21,22,23,24]. For example, S. Bhargava et al. [25] have investigated the UV aging mechanism of waterborne polyurethane coatings. The result showed that there exists scission in functional groups such as C–O–C, C=O, C–H, and CO–NH. Chain scission of the polyurethane binder resulted in the appearance of N–H groups. Boubakri [26] irradiated thermoplastic polyurethane (TPU) coatings for 140 hours of ultraviolet light and showed that glass transition temperature (Tg) of TPU coating decreased in the beginning and then increased with UV-exposure time, then drew the conclusion that there were competition between TPU chain breakage and cross-linking. Liu P et al. [27] have characterized the alkyd coating and polyurethane coating after accelerated UV aging. The results showed that the apparent destruction mode of the alkyd system and the polyurethane system were the same, but the failure mechanism was different. Zhu [28] researched the aging behavior of aliphatic polyurethane coatings and acrylic polyurethane coatings under ultraviolet light irradiation, and concluded that the acrylic urethane was mainly C–O bond cleavage, while the aliphatic urethane was mainly C–N bond cleavage. Rossi [29] showed that the main degradation mechanism of the polyurethane under UV exposure was produced by oxidation of the carbon atom at the alpha position of the nitrogen atom of the urethane group. In conclusion, past research mainly focused on the effects of surface morphology, functional group changes, and the thermodynamic properties of coating.

Polyurea has a unique microphase separation structure composed of a soft segment and a hard segment [26,30,31], of which the soft segment is composed of long aliphatic polyether chains and form amorphous domains whereas the hard segment is composed of a urea bond (NH–CO–NH), forming carbonyl to amino hydrogen bonds [32]. Research indicated that the microphase separation structure of polyurea results in the formation of good physical properties, such as high tensile strength, higher elongation, and so on. Iqbal [33] et al. investigated the effect of soft segment length on their mechanical properties and concluded that the relative content of soft and hard segments had a high correlation with its mechanical strength and modulus. Hydrogen bond plays a pronounced role in defining the macroscopic properties of polyurea, which can promote physical cross-linking between macromolecules [34]. As is known, natural exposure has a vital impact on the macro-properties and constitutive properties of polyurea coating [35,36,37], but there are only a few studies on the effect of exposure in marine atmosphere on the chemical properties of especially hydrogen bond behavior and phase separation morphology.

In this investigation, emphasis was given to the effect of aging on the functional group change, hydrogen bonding behavior, and phase separated morphology in marine atmosphere. The present study evaluated the morphological, chemical, thermal, and mechanical behavior of polyurea during exposure in marine atmosphere and the aging mechanism was analyzed. Spectrometer and contact angle tester, as well as mechanical tests were performed to characterize the degradation. On further studying the SEM, AFM revealed changes in micromorphology. By ATR–FTIR and XPS, changes in the chemical properties of PCB and PCA were analyzed, and emphasis was given to the effect of aging on hydrogen bonding behavior and phase separated morphology.

## 2. Materials and Methods

### 2.1. Materials

#### 2.2.1. Raw Materials

**Figure 1:**
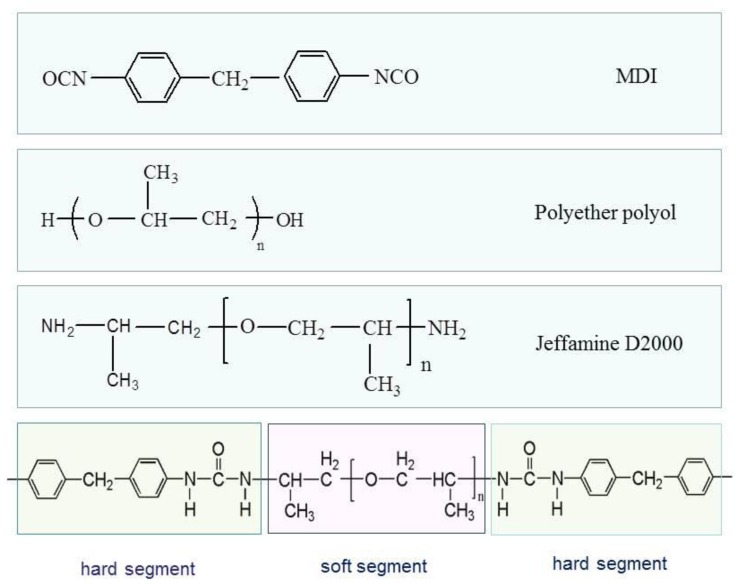
Molecular structures of polyurea precursors.

The polyurea coating was manufactured by Qingdao Shamu Advanced Material Co., Ltd (Qingdao, China), which was a two-component elastomeric material formed by the reaction of component A and B. Component A was a semi-prepolymer of terminal NCO groups formed from diphenylmethane-4,4’ diisocyanate (MDI) and a polyether polyol and component B consisted of a terminal amino polyether Jeffamine^Ⓡ^ D2000 and an amine chain extender. The molecular structures are shown in Figure 1. The two components were, respectively, placed in a raw material tank and mixed, and then thoroughly reacted in a ratio of 1:1 before spraying. The spray equipment was PHX-40 proportioner (PMC Global, Inc., Branford, CA, USA) and AP-2 gun (PMC Global, Inc., Branford, CA, USA). The spray temperature and pressure were 65 °C and 2500 psi, respectively. The coating film thickness was 2 mm with 7 days to be fully cured.

#### 2.2.2. Properties of Polyurea Coating

The polyurea used in this investigation is pure sheet-like coating without spraying on any substrate, and is mainly applied to marine steel structure protection in engineering. It contains zero Volatile Organic Content, which is extremely friendly to the environment. Due to the fast curing speed and insensitivity to moisture and temperature, it can be continuously sprayed on a sloping or vertical surface without sagging or running.

### 2.2. Experimental Method

**Figure 2:**
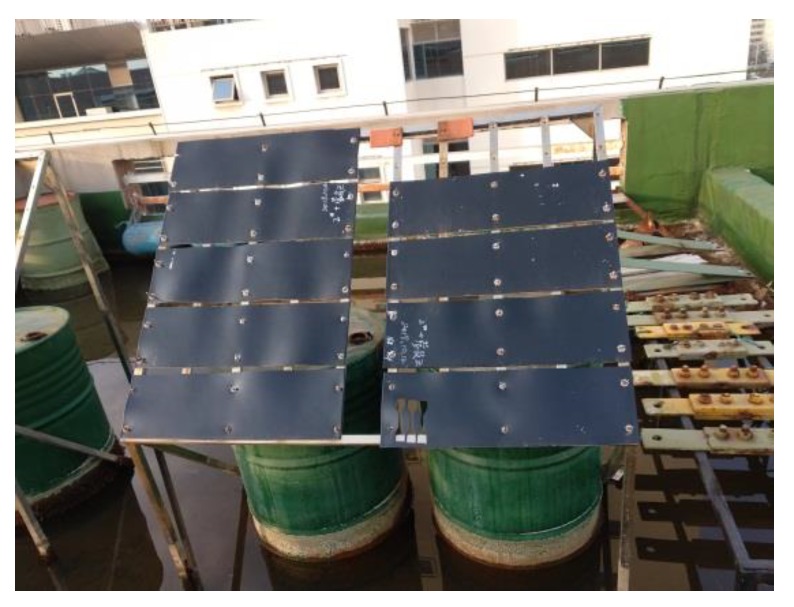
Qingdao University of Technology exposure site.

The experimental site is located at the Qingdao University of Technology, which is situated 4 kilometers away from the actual coastline. Qingdao is situated in the southeastern part of the Shandong Peninsula, borders the Yellow Sea, and is of a temperate monsoon climate, where the annual average relative humidity, temperature, as well as precipitation are 70%, 12.3 °C, and 680 mm, respectively. The polyurea coating used in the experiment should be hung on the exposed frame in the artificial sea pool in accordance with the requirements of GB/T9276-1996 standard. The exposure frame is oriented 45° to the south, ensuring that it is fully exposed to natural sunlight, as shown in Figure 2. The exposure time is 150 days, from mid-March to mid-August.

### 2.3. Characterization

#### 2.3.1. Contact Angle Measurements

Static contact angle measurements were carried out at room temperature on an SDC-200 contact angle goniometer (Dongguan Shengding Precision instrument Co., Ltd., Dongguan, China), using a syringe with a needle to drop a droplet of distilled water on the surface of the sample to be tested, which was then stopped for a few seconds, waiting for the drop to stabilize, and then digital images of the droplet silhouette were captured. The contact angle was measured within 30 s. The polar and dispersive components of the surface energy were calculated using the Fowkes method. Before this measurement, the sample surface before and after aging was ultrasonically cleaned with alcohol to remove impurities and oil.

#### 2.3.2. Mechanical Properties

Tensile properties were conducted using MZ-4000D_1_ universal testing machine (Jiangsu Mingzhu Test Machinery Co., Ltd., Yangzhou, China) at a constant speed of 500 mm/min and at room temperature (25 °C). The dimensions of tensile test samples were taken according to the ASTM D 412 (Standard Test Methods for Vulcanized Rubber and Thermoplastic Elastomers—Tension).

#### 2.3.3. Scanning Electron Microscope

The microstructure of the sample before and after aging was observed by a JSM-7500F scanning electron microscope produced by JEOL (Beijing, China). The resolutions were 1.0 nm (15 kV) and 1.4 nm (1 KV), respectively. The accelerating voltage range was from 0.1KV to 30 KV, and the electron gun used a tungsten filament lamp with magnifications ranging from 25 times to 1,000,000 times. Before the test, the sample was gently wiped with anhydrous ethanol to remove surface dust and was then dried. Then, the sample was sputtered-coated with gold to form a conductive film to improve the image quality and resolution.

#### 2.3.4. Atomic Force Microscopy

Tapping mode atomic force microscopy (AFM) was performed with an Ntegra Prima Scanning Probe system from NT–MDT Prima (NT–MDT Spectrum Instruments Corp., Moscow, Russia). Topographic (height) and phase images were recorded simultaneously under ambient conditions. A silicon cantilever probe with a force constant of 5 N·m^−1^ and a resonance frequency of 70 kHz was used to work on the tapping mode. Before the test, a 15 mm × 15 mm square specimen of PCB and PCA were prepared, and the cleaned samples were obtained by absolute ethanol to remove surface dust.

#### 2.3.5. Thermogravimetric Analysis

Thermogravimetric analysis (TGA) was performed on a STA449C (Netzsch Gerateball, Selb, Germany) under the N_2_ atmosphere at 20 mL/min. In the experiment, a sample weighing approximately 8 mg was heated at 10 °C/min from room temperature to 700 °C.

#### 2.3.6. Attenuated Total Reflectance Fourier Transform Infrared Spectroscopy

ATR–FTIR (Attenuated Total Reflectance) spectra of PCB and PCA were investigated with VERTEX 70 FTIR spectrometer (Bruker Optics, Inc., Ettlingen, Germany) with 32 scans and a resolution of 4 cm^−1^.The spectral region was from 4000 to 500 cm^−1^ and the surface of samples for measurements was cleaned by anhydrous ethanol. The degree of degradation could be detected by detecting changes in the dipole moment associated with the stretching vibration of the functional group.

#### 2.3.7. X-ray Photoelectron Spectroscopic

The X-ray photoelectron spectroscopic (XPS) experiments were performed on Thermo ESCALAB 250XI system (AXIS SUPRA ^DLD^, Shimadzu Kratos Inc., Kyoto, Japan) with Al K_α_ (hv = 1486.6 eV) radiation. The binding energy (BE) scale was regulated by setting the C1s transition at 284.8 eV. The accuracy of the BE values was ± 0.2 eV. The surface of the measurement samples was cleaned by anhydrous ethanol and then naturally dried.

## 3. Results

### 3.1. Surface and Mechanical Properties

**Figure 3:**
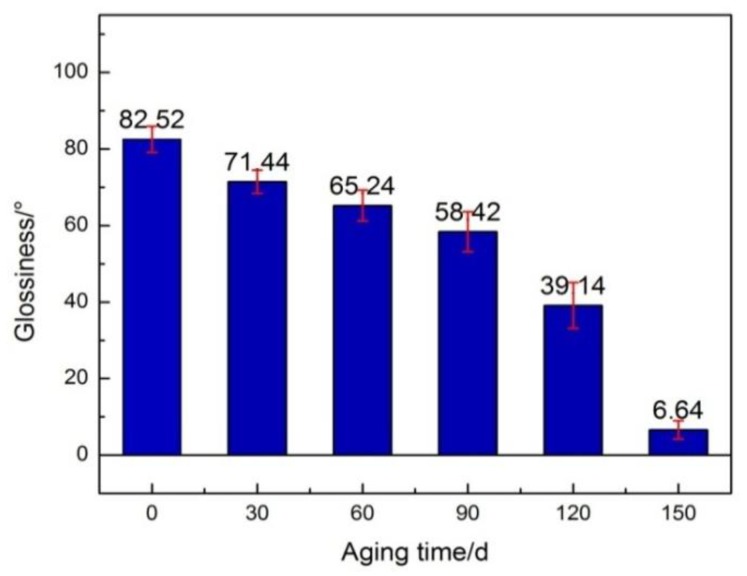
Change in gloss with aging time.

Gloss refers to the ability of a surface to reflect light projected thereon and can be used to indicate the degree of aging of the coating. Figure 3 showed the change of gloss of polyurea coating for 150 days. With extended exposure time, the glossiness value showed a decreasing tendency. As the aging time reached 150 days, the glossiness value decreased 91.95%. Literature [38] stated that the greater the roughness of the coating surface, the lower the reflectivity and gloss value obtained, so the decrease of the coating gloss corresponded well to the cracks of the coating surface.

**Figure 4:**
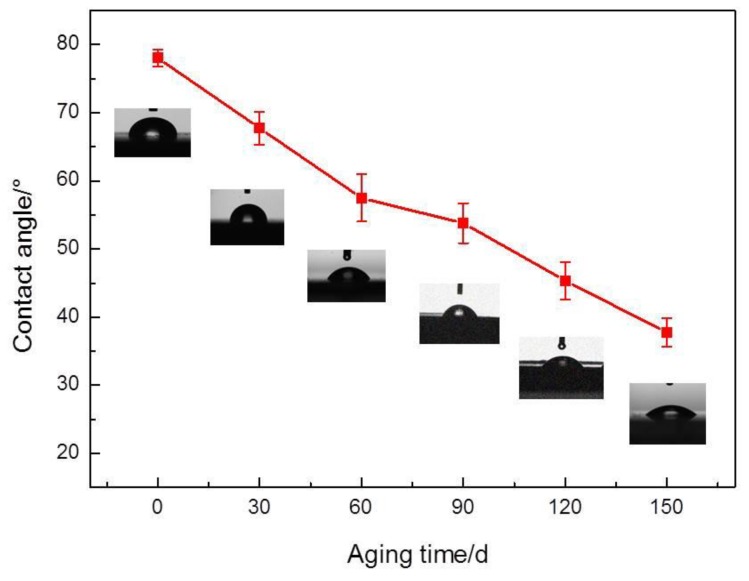
Change in contact angle with aging time.

Determining the water contact angle can provide information about the change in the surface topography through the wetting properties [39,40]. The contact angle results and calculated surface energies of the polyurea coatings were presented in Figure 4. As observed, a steady decrease in the value of the contact angle was evident. The contact angle value of the coating before aging was about 78° ± 1.2° and dropped to 37.7° ± 2.1°, as the aging time reached 150 days, which decreased by about 51.28%. The result indicated a significant change in its wettability on the sample. As the value decreased, the levels of hydrophilicity increased, which could increase the adhesion of contaminants [41]. This was one of reasons for the decrease in gloss.

**Figure 5:**
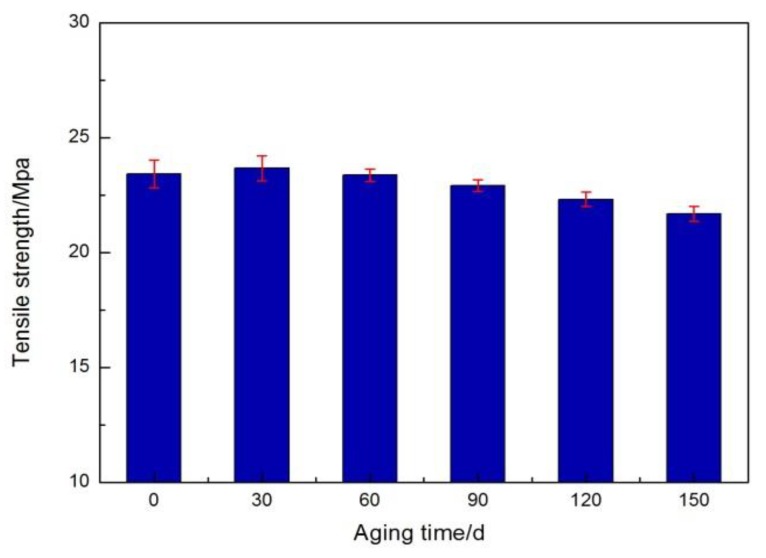
Tensile strength of polyurea coating.

In this section, the aging impact on the tensile properties of the polyurea coating was investigated. Figure 5 showed the result of a polyurea coating during natural exposure to the ocean atmosphere for 150 days. A slight increase in the tensile strength values was observed in the sample during the early stage of aging. This related to the enhancement of molecular cross-linking in the early aging period, and in this period, the effect of molecular cross-linking was greater than molecular bond rupture [26]. However, the tensile strength presented the descent trend on the whole, in the long term, and the tensile strength reduced by 7.44% after exposure for 150 days.

### 3.2. Surface Morphology and Topography

**Figure 6:**
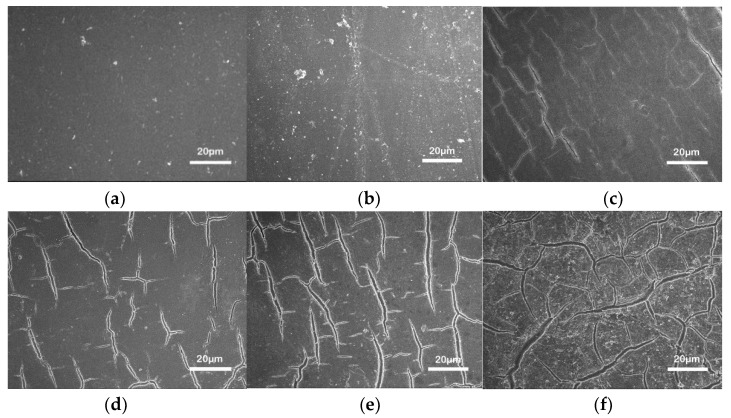
SEM of polyurea—(**a**) 0-day aged; (**b**) 30-days aged; (**c**) 60-days aged; (**d**) 90-days aged; (**e**) 120-days aged; and (**f**) 150-days aged.

**Figure 7:**
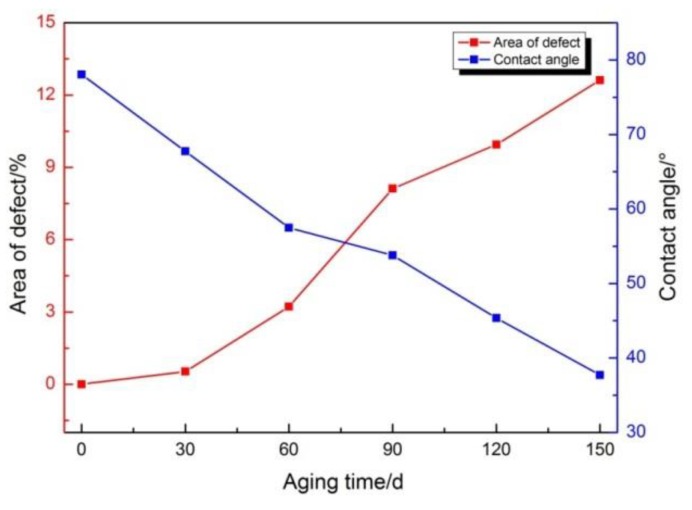
Correlation between the defect area and the contact angle.

The morphology changes on the surface of samples before and after ocean atmosphere exposure for 30 d, 60 d, 90 d, 120 d, and 150 d were obtained by SEM in Figure 6. As shown in Figure 6, the surface of the unaged coating was relatively smooth and homogeneous, and no obvious holes and cracks appeared. With an increase in the aging time, the coating surface generated more cracks, which resulted in the increase of the surface roughness. The defect area calculated by the image J software is shown in Figure 7. The increase in area defects correlated to the exposure duration, which was consistent with research conducted by Youssef [14]. The contact angle decreased with an increase in the defect area, and the two were basically negatively correlated. The appearance of cracks on the coating was related to the breakage of molecular bonds. As found in the literature [38], under the combined action of violet radiation and other influencing factors, the molecules in the coating could degrade to form micropores, and then microcracks had developed. The degradation products might also leave the surface, creating a rough surface.

**Figure 8:**
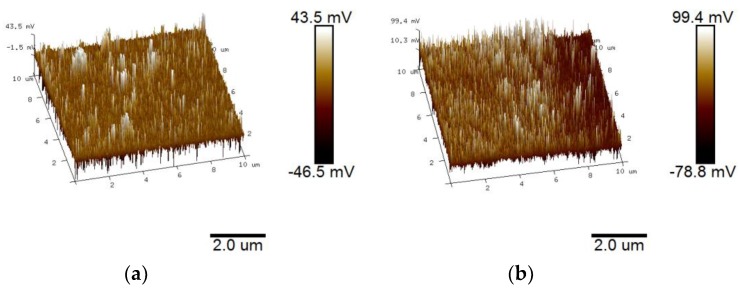
Atomic force microscopy (AFM) of polyurea coating (**a**) 3D phase contrast image of PCB; and (**b**) 3D phase contrast image of PCA.

**Figure 9:**
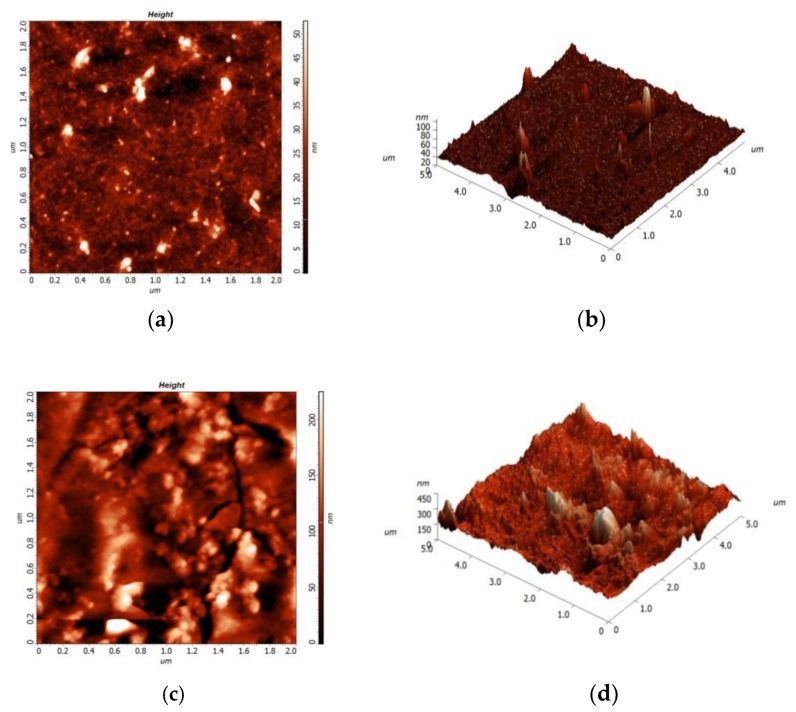

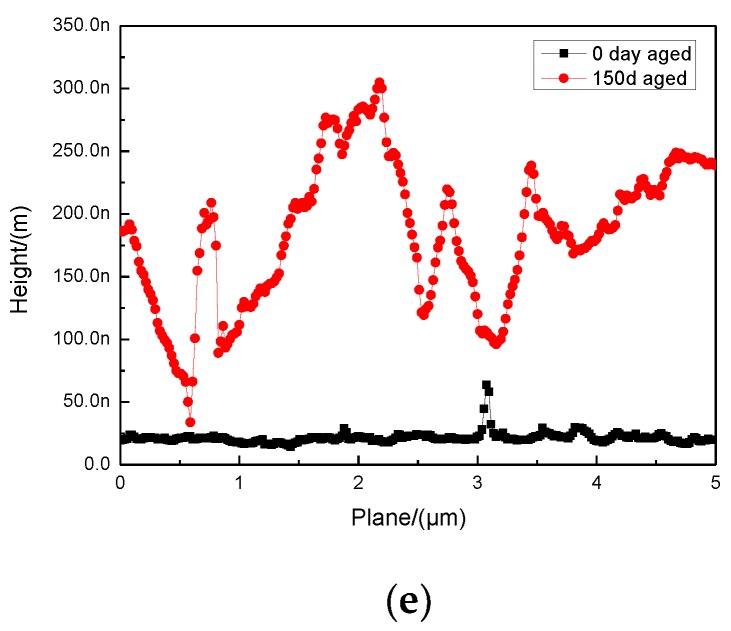
AFM of polyurea coating (**a**) 2D topographic image of PCB; (**b**) 3D topographic image of PCB; (**c**) 2D topographic image of PCA; (**d**) 3D topographic image of PCA; and (**e**) the height of surface before and after 150 d aging.

The surface morphology of PCB and PCA were probed using tapping mode AFM, and the phase contrast, and topographic images were shown in Figure 8 and Figure 9. Figure 8 provided direct visual evidence of two distinct phases—the darker areas correspond to the soft segment domain and the lighter areas correspond to the hard segment domain [42], the hard segment dispersed randomly within the soft segment, forming a microphase separation structure. Compared to the PCB, the PBA had a higher ordinate, indicating a greater degree of microphase separation. Figure 9 showed AFM 2D (Figure 9a,b) and 3D (Figure 9c,d) topographic image representations of PCB and PCA. We can clearly observe in Figure 9c,d, using a qualitative approach, that an increase in surface roughness was achieved after exposure in the marine atmosphere. According to the NOVA software analysis, the root mean square roughness (R_rms_) value for the original sample was 6.03 nm and the value of average roughness (R_a_) was 3.24 nm. The surface topography was relatively flat. As the exposure time reach 150 days, an obvious increase in the roughness degree values could be observed, the R_rms_ value reached 49.50 nm while the R_a_ was close to 36.3 nm. Surface roughness was the main reason for reducing the contact angle [41] which explains the reason for the above-mentioned contact angle reduction. The surface height of PCB and PCA was analyzed by the NOVA software, and the results are shown in Figure 9e. As observed, there are two different roughness profile zones—some very rough zones with a surface height from 50 to 300 nm and other zones had very low roughness values in the range of less than 50 nm.

### 3.3. Thermal Stabilities

**Figure 10:**
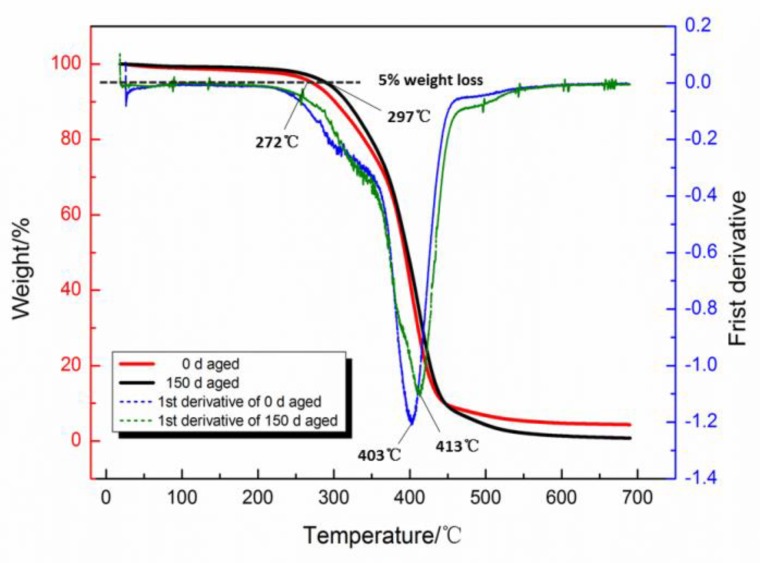
TG and DTG curves for polyurea before and after aging.

Thermal gravity (TG) weight loss curves and the differential thermal gravity (DTG) for PCB and PCA are shown in Figure 10. The onset degradation temperatures are defined by the temperatures of 5% weight loss in TGA curves, whereas temperatures of the maximum degradation rate were evaluated by the peaks in the DTG curves. It can be seen that PCB and PCA both exhibited only one degradation step in nitrogen atmosphere during 250–500 °C. Compared to PCB, PCA had a better thermal stability, for it started to degrade at as high as 297 °C and the maximum weight loss rate occurred at 413 °C, while the coating before aging started to degrade and the maximum weight loss rate occurred at the temperature of 272 °C and 403 °C, respectively. The results of this experiment had the following implications. First, it is possible for PCA to degrade into small molecules, which has a better heat resistance than macromolecules. Second, the soft segment content is reduced, and the coating with a high hard segment content exhibits better heat resistance [42]. Practical reasons need further experimentation.

### 3.4. Chemical Changes

**Figure 11:**
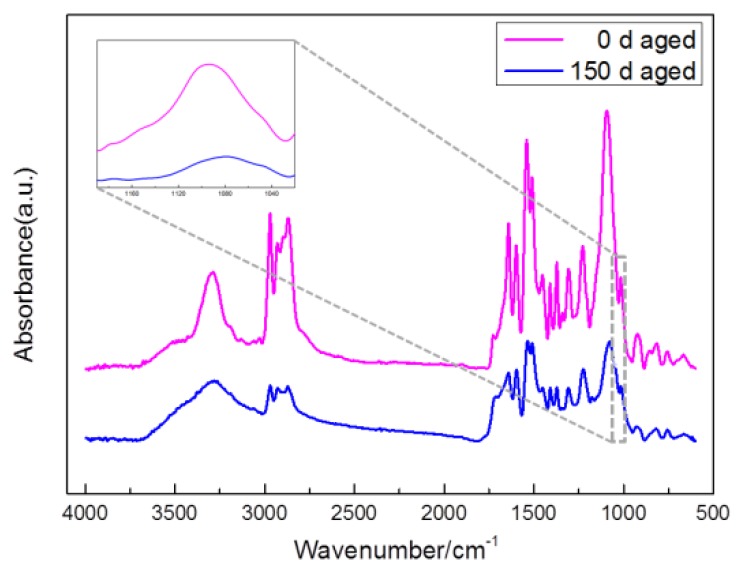
FTIR spectra at the region of 4000–500 cm^−1^ for unaged and aged polyurea.

The FTIR spectra of PCB and PCA are shown in Figure 11. The spectral bands corresponding to the hard segment domains were observed at 3284–3287 cm^−1^ for N–H stretching vibrations and at 1600–1700 cm^−1^ attributed to C=O stretching vibrations. The absorption bands pertaining to the C–N stretching vibrations were at around 1540 cm^−1^, all above were associated with the existence of urea bond in the interior of the composites. The Figure 11 shows that the major difference between the spectra corresponding to PCB and PCA was their intensity of bands. A decrease in the intensity signal of the aged sample indicated the beginning of chain scission or the disappearance of a certain group. For example, the absorbance peaks at 1020–1100 cm^-1^ decreased, confirming the cleavage of the C–O–C bond at exposure aging, and at the same time, the peak of C=O absorption decreased because of the scission of C=O band. The secondary amino N–H stretching vibration peak near 3287 cm^−1^ was weakened and broadened, and shifted towards a lower frequency, which indicated the existence of N–H bond cleavage and the reduction in the degree of hydrogen bonding. The C–H stretching vibration in the 2860–2970 cm^−1^ range was obviously weakened, probably the C–H bond in the D2000 side methyl and methylene had fractured [43]. The results coincided perfectly with the evolution of the surface energy and the tensile properties.

Hydrogen bonding characteristic was one of the most notable features of the polyurea materials, especially polyurea materials with a high hard segment content [44]. As major objectives of hydrogen bonding behavior research, amino, and carbonyl largely determine the hard segment structure and affect the structure and properties of the material [30]. In order to investigate the effect of aging on hydrogen bonding degree, emphasis was given to the amino region and the urea carbonyl region of the spectrum.

The peak at about 3440 cm^−1^ was assigned to the amino that had not formed a hydrogen bond. In the N–H region, the stretching vibration band of N–H of PCB and PCA showed in the frequency of 3287.28 cm^−1^ and 3284.46 cm^−1^, respectively. The magnitude of the shift of the infrared absorption peak number was the indicator of the strength of the hydrogen bond, thus, the length of hydrogen bond can be calculated through the following Equation (1) [45].

(1) $$R=3.21-\frac{\Delta V}{0.548\times 10^{3}}$$

where ∆V represented the shift distance from the amino band without hydrogen bonding to the existing amino band, while R denote the length of hydrogen bond. From the above formula, the lengths of the amino hydrogen bonds before and after aging were both calculated to be about 2.93 Å, thus, aging has little effect on the hydrogen bond length in the N–H region.

A detailed analysis of hydrogen bonding characteristics was carried out by curve fitting the broad band in the carbonyl region of PCB and PCA. The band deconvolution analysis was performed on the carbonyl region at 1620–1690cm^−1^ using the OMNIC 8.2 software (Thermo nicolet, Madison, WI, USA). Hydrogen bonded carbonyl ureas were characterized by the presence of disordered and ordered urea carbonyls, consistent with previous reports [46,47]. The peaks due to the urea domain were characterized by bands at 1626–1645 cm^−1^ and 1651–1669 cm^−1^, attributed to the ordered and disordered hydrogen-bonded urea carbonyls, respectively [43,47,48]. The absorption bands arising around 1673–1685 cm^−1^ corresponded to free hydrogen-bonded urea carbonyl. The wavenumber assignment areas as well as the hydrogen bonding degree of the deconvoluted bands are shown in Table 1. The degree of hydrogen bonding corresponding to the urethane carbonyl was calculated using Equation (2) [47]:

$$X_{o}=\frac{A_{o}}{\left(A_{o}+A_{diso}+A_{free}\right)}$$

(2) $$X_{diso}=A_{diso}/\left(A_{o}+A_{diso}+A_{free}\right)$$

$$X_{b}=X_{o}+X_{diso}$$

where A_o_, A_diso_, and A_free_ denote the area of ordered, disordered, and free hydrogen bonded urea carbonyl, respectively. Whereas, X_o_, X_diso_, and X_b_ showed the percentage of ordered, disordered, and free hydrogen bonding. A comparative analysis of the relative trends in the hydrogen bonding degree of PCB and PCA needed to be considered separately. According to the data in Table 1, major differences between the two sets of samples towards the hydrogen bonding behavior were the degree of ordered, disordered, and total urea hydrogen bonding.

The X_o_ value of PCB and PCA were 57.66% and 46.13%, respectively, whereas the X_b_ values were 83.41% and 74.56%. In contrast to PCB, a significant decrease in the X_o_ and X_b_ values of PCA was observed. The X_o_ value showed a slight increase tendency compared with PCB. Based on the observations above, the ordered hydrogenated urea carbonyl content in PCA was less than PCB, which was related to the intermolecular force, for a reduction in the degree of hydrogen bonding can result in the weakening of the aggregation between hard segments and a decrease in the order of the hard segment structure [49]. Thus, results clearly demonstrated that the intermolecular force of the polyurea after aging was reduced. Literature pointed out [46], the hydrogen bonding in the region of carbonyl can limit the phase separation, as compared to the cases with less hydrogen bonding. The results showed the reduction of hydrogen bonding urea carbonyl content, which improved the degree of microphase separation in the soft and hard sections, in good agreement with the results of AFM.

**Figure 12:**
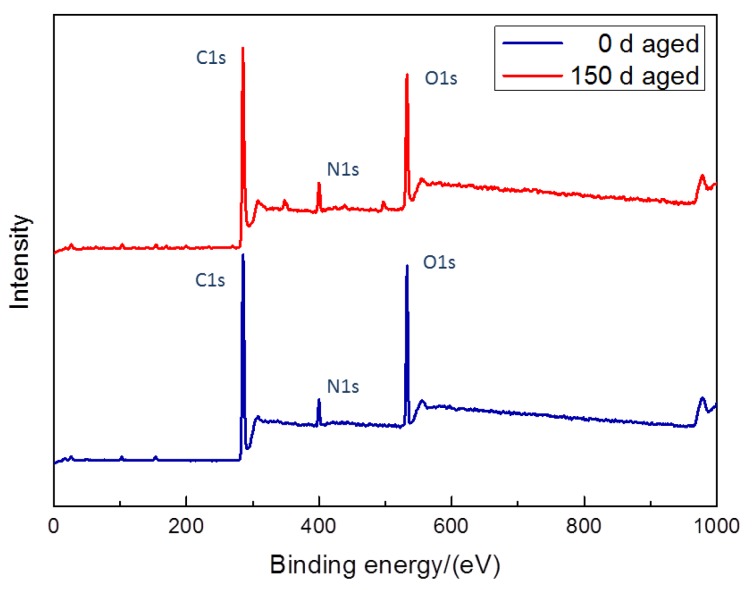
XPS full spectrum analysis before and after polyurea aging.

In the XPS spectrum shown as Figure 12, three obvious signals were detected at 285.3 eV (C 1s), 399.8 eV (N 1s), and 532.7eV (O 1s). It can be seen that relative content of N, C, and O elements was slightly changed and no new elements were produced. The contents of N, C, and O elements and the ratio of elements of PCB and PCA were shown in Table 2. After aging, the relative content of the N element increased more obviously, while the O element and the C element decreased slightly. The oxygen–to–carbon (O/C) and the nitrogen–to–carbon (N/C) ratio of PCB was 0.2598 and 0.0565, respectively, whereas that of PCA was 0.2605 and 0.0765, respectively. The increase of O/C and N/C ratio indicated that the coating degraded during exposure in the marine atmosphere, and the degradation caused the inefficiency of coating.

**Figure 13:**
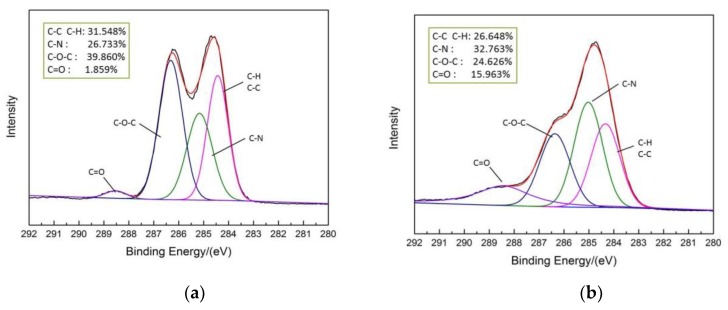
Component-fitted XPS spectra of the C1s peaks of polyurea coating (**a**) before and (**b**) after aging.

In order to elucidate the changes in chemical groups, the C1_S_ spectrum was chosen and deconvolved with the XPSPEAK software as shown in Figure 11. The C1_S_ spectrum of PCB can be resolved into four contributions (Figure 13a). The peak with lowest BE, 284.8 eV, was assigned to carbon atoms which were linked to hydrogen (C–H) or carbon (C–C). The peak observed at 285.7 was assigned to the carbon atom bonded to the nitrogen atom (C–N). The peak located at 286.3 eV corresponded to carbon atoms single-bonded to oxygen atoms (C–O–C) [50]. According to the data in Table 3, the relative content of C–O–C before aging was estimated to be 39.860%, while C=O was about 1.859%. After 150 days of aging, the percentage of C–O–C and C=O content changed greatly, the C–O–C content fell to 24.626%, while the C=O content increased to 15.963%. This phenomenon can be explained as the bond breakage degree of the C–O–C in the soft segment after aging, which was much higher than that of the hard segment urea bond –NHCONH–, and the soft segment phase was more damaged than the hard segment. This indicated the different sensitiveness of the hard and soft segment to the attack of the marine atmosphere exposure, and that the soft segments were more susceptible to erosion, which explained the reason for a greater degree of microphase separation of PCA.

**Scheme 1:**
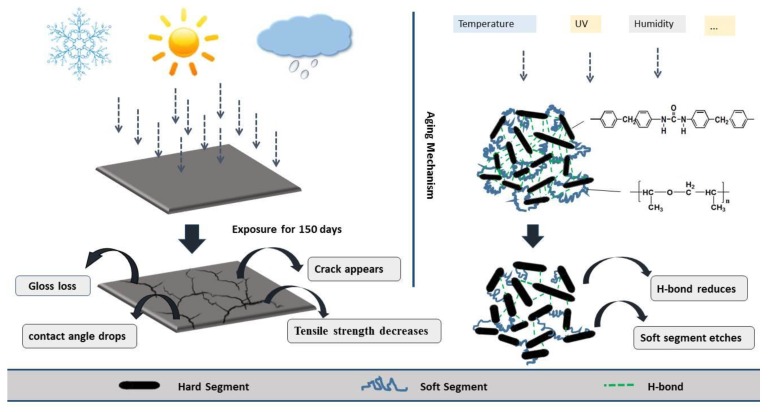
Schematic illustration of the aging mechanism of polyurea coating.

Through the above analysis results, we could draw up the partial aging mechanism shown in Scheme 1. As observed, aging had a great influence on the hydrogen bonding of the hard segment, especially the carbonyl region. After aging, the degree of hydrogen bonding in the carbonyl region was significantly reduced. The decrease of hydrogen bonding degree could weaken the aggregation between hard segments and reduce the order of the hard segment structure, which resulted in the decrease in the mechanical properties. Further studies found that the sensitivity of polyurea between soft and hard segment were significantly different, and the degree of C–O–C bond breakage in the soft segment was higher than that in the hard segment, which caused the soft segment to be etched, and then resulted in the increase of roughness and the microphase separation degree. Macroscopically, the gloss of the material was decreased, and the micro-cracks were generated on the surface of the coating on the microscopic surface, which was one of the important reasons for the decline in the mechanical properties.

## 4. Conclusions

1. An experimental investigation was designed and conducted to determine the aging behavior and mechanism of polyurea coating in marine atmosphere. From the appearance and morphology, PCA exhibits macroscopic phenomena such as loss of light and pulverization while the gloss and contact angle decreased by 91.95% and 51.28%, respectively. It was found that the cracks increased with exposure duration, and the area of defects was basically negatively correlated with the contact angle. The surface roughness of PCA increased significantly, the R_rms_ value reached 49.50 nm, while the R_a_ was close to 36.3 nm.
2. FTIR showed that there was a lot of decrease in functional groups such as C–O–C, C=O and C–N bond. The length of the hydrogen bond in the amide-to-amino region remained stable, via calculation, PCB and PCA were both about 2.93 Å. The total hydrogen bonding degree of the urea carbonyl group decreased from 83.41% to 74.56%, indicating that the interaction between the polyurea molecules was weakened.
3. XPS showed the percentage of C–O–C and C=O content changed greatly, the C–O–C content fell to 24.626%, while the C=O content increased to 15.963%. The soft segment of the PCA was etched more than the hard segment, which increased the degree of microphase separation.

## Figures and Tables

**Table 1:** Degree of hydrogen bonding in the polyurea obtained from the deconvolution of the carbonyl stretching region.

Aging Time/d	Wavenumber (cm^−1^)	Assignment	Area	Percentage of Hydrogen Bonding (%)		
				Xo	Xdiso	Xb
0	1629–1644	Ordered hydrogen bonding	6.9697	57.66		83.41
	1651–1669	Disordered hydrogen bonding	3.1131		25.75	
	1681	free	2.0055			
150	1626–1645	Ordered hydrogen bonding	3.1411	46.13		74.56
	1651–1666	Disordered hydrogen bonding	1.9355		28.43	
	1673–1685	free	1.7321			

**Table 2:** Elemental composition and atomic ratios of polyurea before and after aging.

Coating Treatment	Atomic Percentage/%			Atomic Ratio	
	C	N	O	O/C	N/C
Before the aging	75.97	4.29	19.74	0.2589	0.0565
After aging 150 d	74.81	5.69	19.49	0.2605	0.0761

**Table 3:** Component-Fitted C1s Spectra of polyurea before and after aging.

Element	C–C C–H	C–N	C–O–C	C=O
BE(eV)	284.8	285.7	286.3	288.5
PCB/%	31.548	26.733	39.860	1.859
PCA/%	26.648	32.763	24.626	15.963

## References

[1] Weibo H. (2005). Spray Polyurea Elastomer Technology.

[2] Iqbal N., Tripathi M., Parthasarathy S., Kumar D., Roy P.K. (2016). Polyurea coatings for enhanced blast-mitigation: A review. RSC Adv..

[3] Ma S., Van E.H., Noordover B., Sablong R., Van R.B., Koning C. (2018). Isocyanate-free approach to water-borne polyurea dispersions and coatings. ChemSusChem.

[4] Chattopadhyay D.K., Raju K.V.S.N. (2007). Structural engineering of polyurethane coatings for high performance applications. Prog. Polym. Sci..

[5] Grujicic M., Pandurangan B., He T., Cheeseman B.A., Yen C.F., Randow C.L. (2010). Computational investigation of impact energy absorption capability of polyurea coatings via deformation-induced glass transition. Mater. Sci. Eng..

[6] Gupta V., Youssef G. (2014). Orientation-Dependent Impact Behavior of Polymer/EVA Bilayer Specimens at Long Wavelengths. Exp. Mech..

[7] Davies P., Evrard G. (2007). Accelerated ageing of polyurethanes for marine applications. Polym. Degrad. Stab..

[8] Olajire A.A. (2018). Recent advances on organic coating system technologies for corrosion protection of offshore metallic structures. J. Mol. Liq..

[9] Youssef G., Gupta V. (2012). ARTICLES Dynamic tensile strength of polyurea. J. Mater. Res..

[10] Grujicic M., He T., Pandurangan B. (2011). Development and parameterization of an equilibrium material model for segmented polyurea. Multidiscip. Model. Mater. Struct..

[11] Jain A., Youssef G., Gupta V. (2013). Dynamic tensile strength of polyurea-bonded steel/E-glass composite joints. J. Adhes. Sci. Technol..

[12] Youssef G., Brinson J., Whitten I. (2017). The Effect of Ultraviolet Radiation on the Hyperelastic Behavior of Polyurea. J. Polym. Environ..

[13] Sarva S.S., Deschanel S., Boyce M.C., Chen W. (2007). Stress—Strain behavior of a polyurea and a polyurethane from low to high strain rates. Polymer.

[14] Youssef G., Whitten I. (2016). Dynamic properties of ultraviolet-exposed polyurea. Mech. Time Depend. Mater..

[15] Whitten I., Youssef G. (2016). The effect of ultraviolet radiation on ultrasonic properties of polyurea. Polym. Degrad. Stab..

[16] Youssef G.H. (2011). Dynamic Properties of Polyurea. Ph.D. Thesis.

[17] Youssef G., Gupta V. (2012). Dynamic response of polyurea subjected to nanosecond rise-time stress waves. Mech. Time Depend. Mater..

[18] Amrollahi M., Sadeghi G.M.M. (2016). Assessment of adhesion and surface properties of polyurethane coatings based on non-polar and hydrophobic soft segment. Prog. Org. Coat..

[19] Malviya A.K., Tambe S.P., Malviya A.K., Tambe S.P. (2017). Accelerated test methods for evaluation of WB coating system comprising of epoxy primer and polyurethane top coat. Prog. Color Color. Coat..

[20] Yang X.F., Tallman D.E., Bierwagen G.P., Croll S.G., Rohlik S. (2002). Blistering and degradation of polyurethane coatings under different accelerated weathering tests. Polym. Degrad. Stab..

[21] Liu K., Su Z., Miao S., Ma G., Zhang S. (2016). UV-curable enzymatic antibacterial waterborne polyurethane coating. Biochem. Eng. J..

[22] Wang M., Xu X., Ji J., Yang Y., Shen J., Ye M. (2016). The hygrothermal aging process and mechanism of the novolac epoxy resin. Compos. Part B.

[23] Aglan H., Calhoun M., Allie L. (2008). Effect of UV and hygrothermal aging on the mechanical performance of polyurethane elastomers. J. Appl. Polym. Sci..

[24] Guo T.X., Weng X.Z. (2019). Evaluation of the freeze-thaw durability of surface-treated airport pavement concrete under adverse conditions. Constr. Build. Mater..

[25] Bhargava S., Kubota M., Lewis R.D., Advani S.G., Prasad A.K., Deitzel J.M. (2015). Ultraviolet, water, and thermal aging studies of a waterborne polyurethane elastomer-based high reflectivity coating. Prog. Org. Coat..

[26] Boubakri A., Guermazi N., Elleuch K., Ayedi H.F. (2010). Study of UV-aging of thermoplastic polyurethane material. Mater. Sci. Eng..

[27] Liu P. (2012). Artificial Aging Test Analysis of Alkyd and Polyurethane Coating System. Adv. Mater. Res..

[28] Zhu Y., Xiong J., Tang Y., Zuo Y. (2010). EIS study on failure process of two polyurethane composite coatings. Prog. Org. Coat..

[29] Rossi S., Fedel M., Petrolli S., Deflorian F. (2016). Accelerated weathering and chemical resistance of polyurethane powder coatings. J. Coat. Technol. Res..

[30] Yılgör E., Yılgör I., Yurtsever E. (2002). Hydrogen bonding and polyurethane morphology. I. Quantum mechanical calculations of hydrogen bond energies and vibrational spectroscopy of model compounds. Polymer.

[31] Boubakri A., Elleuch K., Guermazi N., Ayedi H.F. (2009). Investigations on hygrothermal aging of thermoplastic polyurethane material. Mater. Des..

[32] Iqbal N., Kumar D., Roy P.K. (2018). Emergence of time-dependent material properties in chain extended polyureas. J. Appl. Polym. Sci..

[33] Iqbal N., Tripathi M., Parthasarathy S., Kumar D., Roy P.K. (2018). Tuning the properties of segmented polyurea by regulating soft-segment length. J. Appl. Polym. Sci..

[34] Iqbal N., Tripathi M., Parthasarathy S., Kumar D., Roy P.K. (2018). Aromatic versus Aliphatic: Hydrogen Bonding Pattern in Chain-Extended High-Performance Polyurea. ChemistrySelect.

[35] Zhang X., Wang J., Guo W., Zou R. (2017). A bilinear constitutive response for polyureas as a function of temperature, strain rate and pressure. J. Appl. Polym. Sci..

[36] Jia Z., Amirkhizi A.V., Nantasetphong W., Nemat-Nasser S. (2016). Experimentally-based relaxation modulus of polyurea and its composites. Mech. Time Depend. Mater..

[37] Chao L., Ma C., Xie Q., Zhang G. (2017). Self-repairing silicone coating for marine anti-biofouling. J. Mater. Chem. A.

[38] Zhang H., Dun Y., Tang Y., Zuo Y., Zhao X. (2016). Correlation between natural exposure and artificial ageing test for typical marine coating systems. J. Appl. Polym. Sci..

[39] Zhang Y., Qi Y., Zhang Z. (2016). Synthesis of PPG-TDI-BDO polyurethane and the influence of hard segment content on its structure and antifouling properties. Prog. Org. Coat..

[40] Meiron T.S., Marmur A., Saguy I.S. (2004). Contact angle measurement on rough surfaces. J. Colloid Interface Sci..

[41] Huang F., Wei Q., Wang X., Xu W. (2006). Dynamic contact angles and morphology of PP fibres treated with plasma. Polym. Test..

[42] Awad W.H., Wilkie C.A. (2010). Investigation of the thermal degradation of polyurea: The effect of ammonium polyphosphate and expandable graphite. Polymer.

[43] Ping L. (2007). Studies on the Novel Polyaspartic Ester Based Polyurea Coatings for Marine Concrete Protection. Ph.D. Thesis.

[44] Lu P., Chen G.H., Huang W.B. (2007). Degradation of Polyaspartic Polyurea Coating under Different Accelerated Weathering Tests. J. Sichuan Univ. Eng. Sci. Ed..

[45] Lu P., Chen G., Huang W. (2008). Study on Synthesis, Morphology and Properties of Novel Polyureas Based on Polyaspartic Esters. J. Chem. Eng. Chin. Univ..

[46] Jiang B., Tsavalas J.G., Sundberg D.C. (2018). Morphology control in surfactant free polyurethane/acrylic hybrid lattices—The special role of hydrogen bonding. Polymer.

[47] Chavan J.G., Rath S.K., Praveen S., Kalletla S., Patri M. (2016). Hydrogen bonding and thermomechanical properties of model polydimethylsiloxane based poly(urethane-urea) copolymers: Effect of hard segment content. Prog. Org. Coat..

[48] Rakshit P., Joshi N., Jain R., Shah S. (2016). Synthesis and Characterization of Polyurea Resin for Dielectric Coating Applications. Polym. Plast. Technol. Eng..

[49] Yang J., Wang G., Hu C. (2006). Effect of Chain Extenders on Structures and Properties of Aliphatic Polyurethaneureas and Polyureas. Acta Chim. Sin..

[50] Chen T.F., Siow K.S., Ng P.Y., Nai M.H., Lim C.T., Yeop Majlis B. (2016). Ageing properties of polyurethane methacrylate and off-stoichiometry thiol-ene polymers after nitrogen and argon plasma treatment. J. Appl. Polym. Sci..

